# The phosphatidylinositol (4,5)-bisphosphate-Rab35 axis regulates migrasome formation

**DOI:** 10.1038/s41422-023-00811-5

**Published:** 2023-05-04

**Authors:** Tianlun Ding, Jinyao Ji, Weiying Zhang, Yuheng Liu, Boqi Liu, Yiyang Han, Chunlai Chen, Li Yu

**Affiliations:** 1 State Key Laboratory of Membrane Biology, Tsinghua University-Peking University Joint Center for Life Sciences, Beijing Frontier Research Center for Biological Structure, School of Life Sciences, Tsinghua University, Beijing, China; 2 School of Life Sciences, Beijing Advanced Innovation Center for Structural Biology, Beijing Frontier Research Center of Biological Structure, Tsinghua University, Beijing, China; 3 State Key Laboratory of Membrane Biology, Tsinghua University-Peking University Joint Center for Life Sciences, School of Life Sciences, Tsinghua University, Beijing, China

**Keywords:** Cell adhesion, Cell migration

## Abstract

Migrasomes are recently discovered organelles, which are formed on the ends or branch points of retraction fibers at the trailing edge of migrating cells. Previously, we showed that recruitment of integrins to the site of migrasome formation is essential for migrasome biogenesis. In this study, we found that prior to migrasome formation, PIP5K1A, a PI4P kinase which converts PI4P into PI(4,5)P_2_, is recruited to migrasome formation sites. The recruitment of PIP5K1A results in generation of PI(4,5)P_2_ at the migrasome formation site. Once accumulated, PI(4,5)P_2_ recruits Rab35 to the migrasome formation site by interacting with the C-terminal polybasic cluster of Rab35. We further demonstrated that active Rab35 promotes migrasome formation by recruiting and concentrating integrin α5 at migrasome formation sites, which is likely mediated by the interaction between integrin α5 and Rab35. Our study identifies the upstream signaling events orchestrating migrasome biogenesis.

## Introduction

Migrasomes are vesicular organelles which form on retraction fibers at the trailing edge of migrating cells.^1^ Migrasomes have important physiological functions including organ morphogenesis,^2^ mitochondrial quality control^3^ and lateral transfer of protein and mRNA between cells.^4^ During migrasome formation, integrins are first targeted to the ends or branch points of retraction fibers to form integrin foci. These foci will later grow into migrasomes and are operationally defined as migrasome formation sites.^5^ Once the integrin foci are formed, tetraspanin-enriched microdomains start to assemble at the migrasome formation site, and eventually expand into migrasomes.^6^ How integrins are targeted to migrasome formation sites is currently unknown.

Organelle biogenesis is a highly orchestrated process. Phosphoinositides are lipid signaling molecules which play a central role in organelle biogenesis. For example, during autophagosome formation, phosphatidylinositol 3-monophosphate (PI3P)-enriched structures (named omegasomes) are first formed on the endoplasmic reticulum (ER), which then serve as a platform to recruit proteins essential for autophagosome formation.^7^ It is currently unclear whether migrasome formation is a regulated process which involves signaling pathway(s).

Phosphatidylinositol (4,5)-bisphosphate (PI(4,5)P_2_) is a multi-functional lipid which regulates a large array of subcellular processes. PI(4,5)P_2_ is the most abundant phosphoinositide and is mainly localized in the plasma membrane.^8^ It is commonly believed that the majority of cellular PI(4,5)P_2_ is synthesized by phosphatidylinositol 4-monophosphate 5-kinases (PIP5Ks), which convert phosphatidylinositol 4-monophosphate (PI4P) to PI(4,5)P_2_.^9^ In many cases, PI(4,5)P_2_ carries out its functions through interaction with its partner proteins. So far, multiple PI(4,5)P_2_ interaction domains, including PH, ANTH, ENTH and FERM, have been identified.^10^

In this study, we demonstrate that the biogenesis of migrasomes is a highly regulated process in which PI(4,5)P_2_ signaling plays a central role. We found that prior to migrasome formation, PI(4,5)P_2_ is synthesized de novo at migrasome formation sites by PIP5K1A, and migrasome formation is blocked when PI(4,5)P_2_ generation is inhibited. We identified Rab35 as a migrasome-localized PI(4,5)P_2_-binding protein. Further study revealed that Rab35 is essential for migrasome formation. Mechanistically, Rab35 is recruited to migrasome formation sites via interaction with PI(4,5)P_2_. Subsequently, through Rab35–integrin α5 interaction, Rab35 recruits integrin α5 to migrasome formation sites, which prepares the sites for tetraspanin-dependent expansion.

## Results

### Generation of PI(4,5)P_2_ by PIP5K1A at the migrasome formation sites

**Fig. 1:**
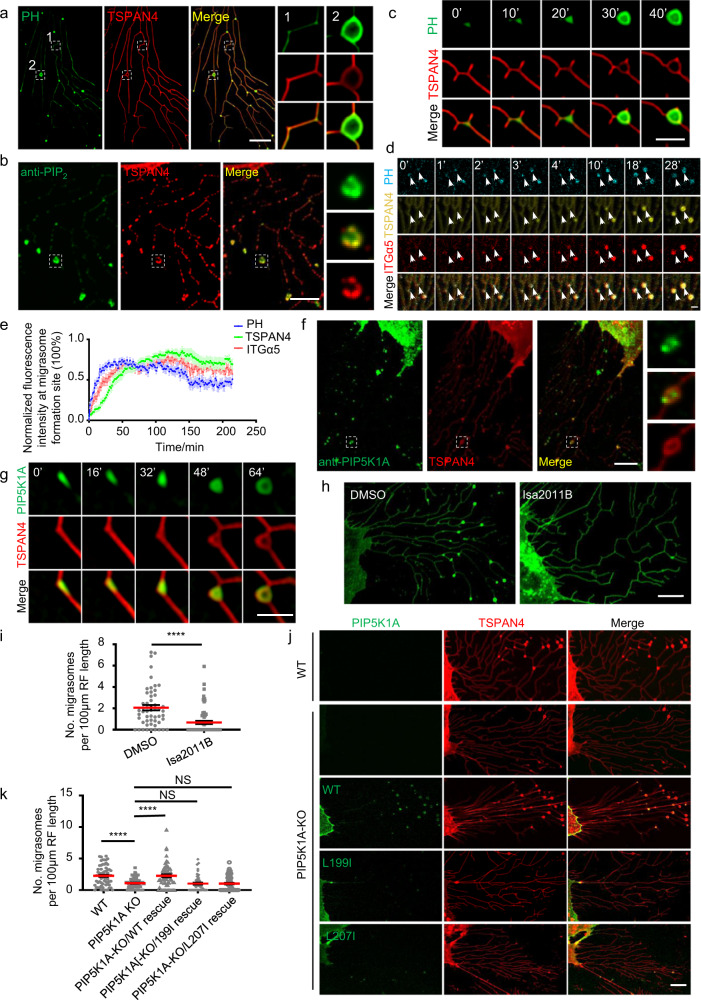
Generation of PI(4,5)P_2_ by PIP5K1A at the migrasome formation sites. **a** Live-cell structure illumination microscopy (SIM) images of NRK cells expressing PH-GFP and TSPAN4-mCherry. Green, PH; red, TSPAN4; yellow, merge. Scale bar, 10 μm. Boxed regions are enlarged on the right. **b** Immunofluorescence imaging of PI(4,5)P_2_ in an NRK cell line overexpressing TSPAN4-mCherry. Cells were imaged by confocal microscopy. Green, PI(4,5)P_2_; red, TSPAN4; yellow, merge. Scale bar, 10 μm. Boxed regions are enlarged on the right. **c** Time-lapse imaging of NRK cells stably expressing PH-GFP and TSPAN4-mCherry. Images were captured every 10 min by SIM. Green, PH; red, TSPAN4; yellow, merge. Scale bar, 2 μm. **d** Time-lapse imaging of NRK cells stably expressing PH-TagBFP, TSPAN4-GFP and ITGα5-mCherry. Confocal microscopy images were captured every 1 min. Cyan, PH; yellow, TSPAN4; red, ITGα5; white, merge. Scale bar, 2 μm. White arrowheads indicate two migrasome formation sites. **e** Statistical analysis of normalized fluorescence intensity of PH, TSPAN4 and ITGα5 at migrasome formation sites during migrasome formation. Data are presented as mean ± SEM; *n* = 10 from four independent cells. **f** Immunofluorescence imaging of PIP5K1A in the NRK cell line overexpressing TSPAN4-mCherry. Cells were imaged by confocal microscopy. Green, PIP5K1A; red, TSPAN4; yellow, merge. Scale bar, 10 μm. **g** Time-lapse imaging of NRK cells stably expressing PIP5K1A-GFP and TSPAN4-mCherry. Images were captured every 7 min by SIM. Green, PIP5K1A; red, TSPAN4; yellow, merge. Scale bar, 2 μm. **h** Live-cell images of NRK cells expressing TSPAN4-GFP under treatment with ISA2011B or DMSO (control). Cells were imaged by confocal microscopy. Green, TSPAN4. Scale bar, 10 μm. **i** Statistical analysis of the number of migrasomes per 100 μm retraction fiber per cell. The original images were captured as in **h**. *n* = 54 for control; *n* = 64 for ISA011B treatment. Data are presented as mean ± SEM; unpaired *t*-test. **j** Live-cell confocal microscopy images of WT NRK-TSPAN4-mCherry cells and PIP5K1A-KO NRK-TSPAN4-mCherry cells without and with stable expression of GFP-PIP5K1A(WT), GFP-PIP5K1A(L199I) and GFP-PIP5K1A(L207I). Green, PIP5K1A; red, TSPAN4; yellow, merge. Scale bar, 10 μm. **k** Statistical analysis of the number of migrasomes per 100 μm retraction fiber per cell. The original images were captured as in **j**. *n* = 68 for WT; *n* = 61 for PIP5K1A KO; *n* = 65 for PIP5K1A-KO/WT rescue; *n* = 66 for PIP5K1A-KO/D306A rescue; *n* = 60 for PIP5K1A-KO/L199I rescue; *n* = 63 for PIP5K1A-KO/L207I rescue. Data are presented as mean ± SEM; unpaired *t*-test.

Previously, we found that PLCγ-PH-GFP, a probe for PI(4,5)P_2_, can label migrasomes, which we confirmed here (Fig. 1a). Staining of cells using an anti-PI(4,5)P_2_ antibody also showed enrichment of the PI(4,5)P_2_ signal in migrasomes (Fig. 1b). These results suggest that migrasomes contain PI(4,5)P_2_. To study the dynamics of PI(4,5)P_2_ on migrasomes, we carried out time-lapse imaging. We found that PLCγ-PH-GFP was recruited to migrasomes before TSPAN4 (Fig. 1c; Supplementary information, Video S1). Previously we reported that prior to recruitment of TSPAN4, integrin α5 forms foci on retraction fibers which define the sites for migrasome formation. Next, we examined the dynamics of PLCγ-PH-TagBFP compared to that of integrin α5. We found that the recruitment of PLCγ-PH-TagBFP was slightly faster than that of integrin α5 (Fig. 1d, e). Together, these data suggest that PI(4,5)P_2_ is generated on or recruited to the migrasome formation site prior to migrasome growth.

PI(4,5)P_2_ can be generated by PI4P kinase, which converts PI4P into PI(4,5)P_2_. To test whether PI4P kinase is involved in generation of PI(4,5)P_2_ at the site of migrasome formation, we stained cells with an antibody against PIP5K1A, the major isoform of PI4P kinase expressed in NRK cells. Indeed, we found that PIP5K1A was localized on migrasomes (Fig. 1f). Similarly, ectopically expressed PIP5K1A-GFP was localized on migrasomes, and time-lapse imaging showed that PIP5K1A-GFP was recruited to the site of migrasome formation prior to the recruitment of TSPAN4 (Fig. 1g; Supplementary information, Video S2). This is consistent with the appearance of the PI(4,5)P_2_ signal (Fig. 1c).

Next, we tested whether PIP5K1A is responsible for PI(4,5)P_2_ generation at the sites of migrasome formation. We treated cells with ISA2011B, a PIP5K1A inhibitor. We found that ISA2011B treatment blocked migrasome formation (Fig. 1h, i). To further confirm that PI(4,5)P_2_ is required for migrasome formation, we generated PIP5K1A knockout (KO) cells, and found that indeed formation of migrasomes was markedly reduced (Fig. 1j, k; Supplementary information, Fig. S1). Ectopically expressing wild-type (WT) PIP5K1A, but not two PIP5K1A mutants with reduced kinase activity, restored migrasome formation (Fig. 1j, k; Supplementary information, Fig. S2). This suggests that the enzyme activity of PIP5K1A is required for migrasome formation, and provides further evidence that the level of PI(4,5)P_2_ is a determinant of migrasome formation. It is worth noting that KO of PIP5K1A did not affect retraction fiber formation or cell migration (Supplementary information, Fig. S3a and Videos S3, S4). Together, these results suggest that generation of PI(4,5)P_2_ by PIP5K1A at the site of migrasome formation is required for migrasome biogenesis.

Since PI(4,5)P_2_ can be hydrolyzed by lipid phosphatases, we next examined the localization of the known PI(4,5)P_2_ phosphatases. We found that PLCD3 was localized on migrasomes (Supplementary information, Fig. S3b). To further test the role of PI(4,5)P_2_ in migrasome formation, we generated a PLCD3 KO cell line. In this cell line, the formation of migrasomes was significantly enhanced (Supplementary information, Fig. S3c, d), and ectopic expression of PLCD3 restored migrasome formation to the normal level. Together, these data provide further evidence to support the role of PI(4,5)P_2_ in migrasome formation.

### PI(4,5)P_2_ recruits Rab35 to migrasome formation sites

**Fig. 2:**
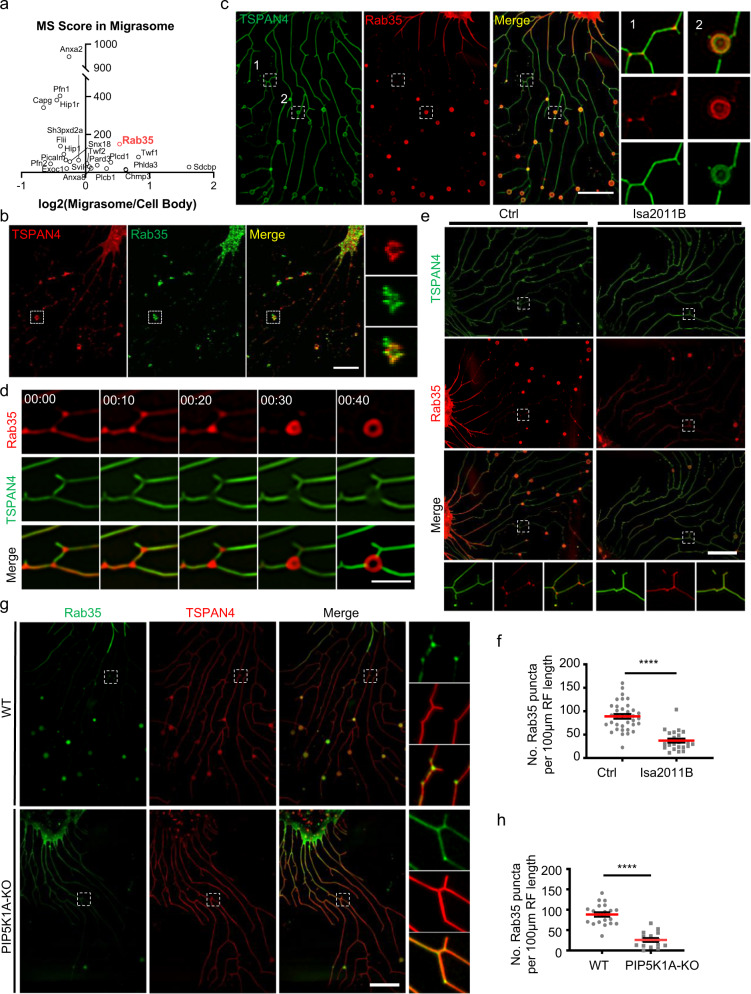
PI(4,5)P_2_ recruits Rab35 to migrasome formation sites. **a** MA plot of PI(4,5)P_2_-binding proteins in migrasomes compared to cell bodies. Rab35 is highlighted in red. **b** Immunofluorescence imaging of Rab35 in NRK cells expressing TSPAN4-mCherry. Cells were imaged by confocal microscopy. Green, Rab35; red, TSPAN4; yellow, merge. Scale bar, 10 μm. Boxed regions are enlarged on the right. **c** Live-cell SIM images of NRK cells stably expressing TSPAN4-GFP and mCherry-Rab35. Green, TSPAN4; red, Rab35; yellow, merge. Scale bar, 10 μm. Boxed regions are enlarged on the right. **d** Time-lapse imaging of NRK cells stably expressing TSPAN4-GFP and mCherry-Rab35. Images were captured every 10 min by SIM. Green, TSPAN4; red, Rab35; yellow, merge. Scale bar, 2 μm. **e** Live-cell SIM images of NRK cells stably expressing TSPAN4-GFP and mCherry-Rab35. Cells were treated without (Ctrl) or with ISA2011B for 8 h. Green, TSPAN4; red, Rab35; yellow, merge. Scale bar, 10 μm. Boxed regions are enlarged at the bottom. **f** Statistical analysis of the number of Rab35 puncta per 100 μm retraction fiber per cell. The original images were captured as in **f**. *n* = 36 for control; *n* = 23 for ISA2011B treatment. Data are presented as mean ± SEM; unpaired *t*-test. **g** Live-cell SIM images of WT or PIP5K1A-KO NRK cells stably expressing GFP-Rab35 and TSPAN4-mCherry. Green, Rab35; red, TSPAN4; yellow, merge. Scale bar, 10 μm. Enlarged images of boxed regions are shown on the right. **h** Statistical analysis of the number of Rab35 puncta per 100 μm retraction fiber per cell. The original images were captured as in **g**. *n* = 21 for WT; *n* = 16 for PIP5K1A KO. Data are presented as mean ± SEM; unpaired *t*-test.

Next, we investigated how PI(4,5)P_2_ regulates migrasome formation. We reasoned that PI(4,5)P_2_ may regulate migrasome formation by recruiting PI(4,5)P_2_-binding proteins which are required for migrasome formation. To screen the possible migrasome-localized PI(4,5)P_2_-binding proteins, we first compiled a list of all the PI(4,5)P_2_-binding proteins in the rat genome. Next, we compared this list to the list of proteins in migrasomes, which we identified previously by mass spectrometry (MS) analysis of purified migrasomes.^11^ We found 23 PI(4,5)P_2_-binding proteins on the MS list, including Rab35 (Fig. 2a). We then generated mCherry-tagged constructs for 19 of these proteins, and found that some of them were localized on migrasomes (Supplementary information, Fig. S4). Among these proteins, we picked Rab35 for further study, since Rabs play key roles in organelle biogenesis.

We first confirmed the recruitment of Rab35 by staining cells with anti-Rab35 antibody. We found that endogenous Rab35 was indeed localized on migrasomes and on small puncta along the retraction fiber (Fig. 2b; Supplementary information, Fig. S5). Similarly, ectopically expressed mCherry-Rab35 was localized on migrasomes and on migrasome formation sites (Fig. 2c). To study the dynamics of Rab35 recruitment, we carried out time-lapse imaging using mCherry-Rab35 (Fig. 2d). We found that the Rab35 signal was first evenly and diffusely distributed along a retraction fiber. Prior to migrasome formation, the Rab35 signals were gradually concentrated at the branch points and became more intense. Eventually, the Rab35-positive puncta started to enlarge and grew into migrasomes (Fig. 2d; Supplementary information, Video S5). These results suggest that Rab35 is recruited to the sites of migrasome formation prior to migrasome biogenesis.

Next, we tested whether the Rab35 recruitment to the migrasome formation sites is PI(4,5)P_2_ dependent. We treated cells with the PIP5K1A inhibitor ISA2011B, and observed impaired recruitment of Rab35 to migrasome formation sites (Fig. 2e, f). Similarly, Rab35 failed to be recruited to the sites of migrasome formation in PIP5K1A KO cells (Fig. 2g, h). These results confirm that the recruitment of Rab35 is PI(4,5)P_2_ dependent.

### Rab35 is required for migrasome formation

**Fig. 3:**
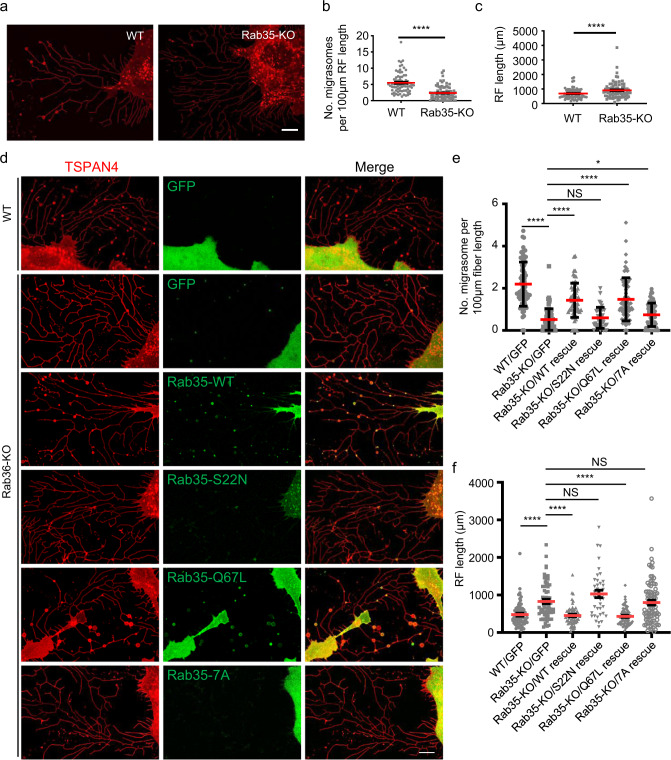
Rab35 promotes migrasome formation. **a** Live-cell confocal microscopy images of WT and Rab35-KO NRK-mCherry-TSPAN4 cells. Red, TSPAN4. Scale bar, 10 μm. **b** Statistical analysis of the number of migrasomes per 100 μm retraction fiber per cell. The original images were captured as in **a**. *n* = 72 for WT; *n* = 80 for Rab35 KO. Data are presented as mean ± SEM; unpaired *t*-test. **c** Statistical analysis of retraction fiber length per cell. The original images were captured as in **a**. *n* = 72 for WT; *n* = 80 for Rab35 KO. Data are presented as mean ± SEM; unpaired *t*-test. **d** Live-cell confocal microscopy images of WT NRK-TSPAN4-mCherry cells with stable expression of GFP and Rab35-KO NRK-TSPAN4-mCherry cells with stable expression of GFP, GFP-Rab35-WT, GFP-Rab35-S22N, GFP-Rab35-Q67L and GFP-Rab35–7A. Green, GFP or GFP-Rab35; red, TSPAN4; yellow, merge. Scale bar, 10 μm. **e** Statistical analysis of the number of migrasomes per 100 μm retraction fiber per cell. The original images were captured as in **d**. *n* = 72 for WT/GFP; *n* = 70 for Rab35-KO/GFP; *n* = 63 for Rab35-KO/WT rescue; *n* = 39 for Rab35-KO/S22N rescue; *n* = 66 for Rab35-KO/Q67L rescue; *n* = 82 for Rab35-KO/7A rescue. Data are presented as mean ± SEM; unpaired *t*-test. **f** Statistical analysis of retraction fiber length per cell. The original images were captured as in **d**. *n* = 72 for WT/GFP; *n* = 70 for Rab35-KO/GFP; *n* = 63 for Rab35-KO/WT rescue; *n* = 39 for Rab35-KO/S22N rescue; *n* = 66 for Rab35-KO/Q67L rescue; *n* = 82 for Rab35-KO/7A rescue. Data are presented as mean ± SEM; unpaired *t*-test.

Next, to test whether Rab35 is required for migrasome formation, we generated a Rab35 KO cell line. We found that KO of Rab35 severely impaired migrasome formation (Fig. 3a, b). Interestingly, KO of Rab35 enhanced the number and length of retraction fibers (Fig. 3a, c). Stable expression of WT Rab35 and constitutively active Rab35-Q67L in the Rab35 KO cell line rescued migrasome formation, while stable expression of a dominant-negative mutant Rab35-S22N failed to rescue migrasome formation (Fig. 3d, e). These results indicate that active Rab35 is required for migrasome formation.

Previous literature showed that PI(4,5)P_2_ recruits Rab35 to the plasma membrane by interacting with its C-terminal polybasic amino acid cluster, which consists of a stretch of positively charged Lys and Arg residues.^12^ When we replaced the polybasic cluster with the non-polar neutral amino acid Ala (Rab35-7A), we found that the mutant Rab35 could not be recruited to migrasomes and failed to rescue migrasome formation (Fig. 3d, e). Together, these data suggest that recruitment of Rab35 to the sites of migrasome formation by PI(4,5)P_2_ is required for migrasome formation.

To further confirm the role of Rab35 in migrasome formation, we established cell lines stably expressing WT Rab35, dominant-negative Rab35-S22N and constitutively active Rab35-Q67L. Consistent with the rescue experiment, we found that overexpressing WT Rab35 and constitutively active Rab35-Q67L enhanced migrasome formation, while expressing dominant-negative Rab35-S22N reduced migrasome formation (Supplementary information, Fig. S6a, b). Moreover, expressing dominant-negative Rab35-S22N enhanced retraction fiber formation (Fig. 3f; Supplementary information, Fig. S6a, c). Together, these data suggest that Rab35 plays an important role in regulation of retraction fiber length and migrasome formation.

### Rab35 promotes migrasome formation by targeting integrin α5 to migrasomes

**Fig. 4:**
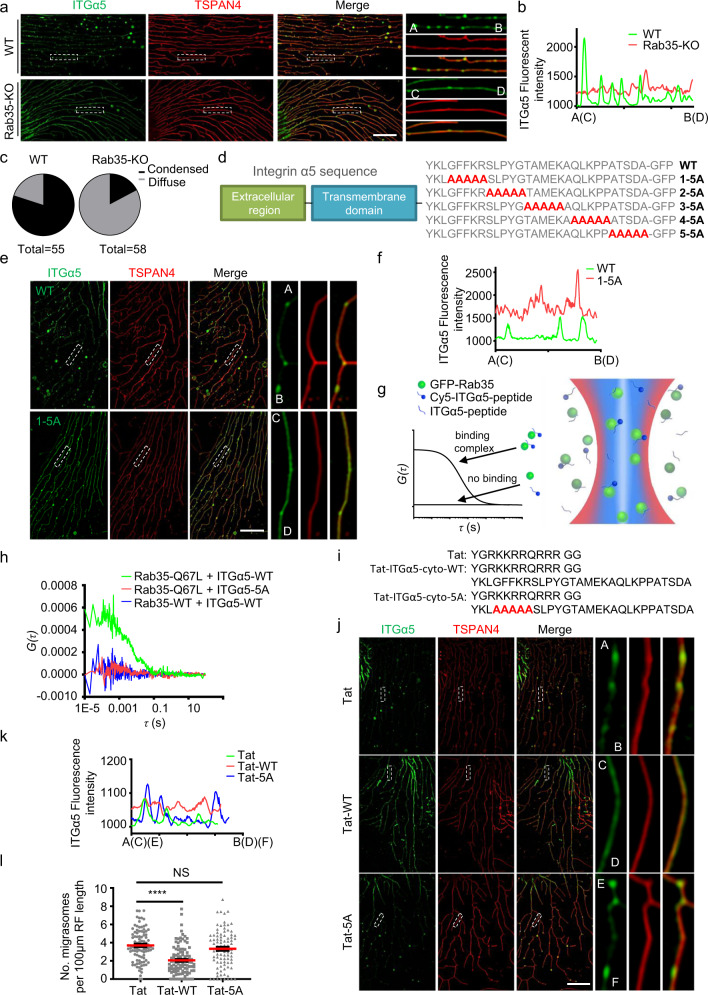
Rab35 recruits integrin to migrasome formation sites. **a** Live-cell SIM images of WT or Rab35-KO NRK-TSPAN4-mCherry cells stably expressing ITGα5-GFP. Green, ITGα5; red, TSPAN4; yellow, merge. Scale bar, 10 μm. Boxed regions are enlarged on the right. **b** The boxed regions from **a** were quantified for the ITGα5-GFP fluorescence intensity. **c** The images from **a** were quantified for the percentage of cells with condensed or diffuse ITGα5-GFP distribution. **d** Schematic representation of ITGα5 showing the amino acid sequences of the cytoplasmic domain in WT and the 5 mutants (1-5A, 2-5A, 3-5A, 4-5A, 5-5A). **e** Live-cell SIM images of NRK-TSPAN4-mCherry cells stably expressing ITGα5 WT or 1-5A. Green, ITGα5; red, TSPAN4; yellow, merge. Scale bar, 10 μm. The boxed regions are enlarged on the right. **f** The boxed regions from **e** were quantified for the ITGα5-GFP fluorescence intensity. **g** Diagram of the dcFCCS assay: GFP-Rab35 (green dots), Cy5-ITGα5-cyto (blue dots with blue lines) and ITGα5-cyto (blue lines) diffuse freely through the confocal detection volume of 488 nm (blue zone) and 640 nm (red zone) lasers. Only complexes containing both GFP and Cy5 fluorescence signals can contribute to cross-correlation curves. **h** dcFCCS curves of 100 nM GFP-Rab35-WT mixed with 500 nM Cy5-ITGα5-cyto-WT (blue); 100 nM GFP-Rab35-Q67L mixed with 500 nM Cy5-ITGα5-cyto-WT (green); and 100 nM GFP-Rab35-Q67L mixed with Cy5-ITGα5-cyto-5A (red). **i** Sequences of Tat peptides. The Tat peptide (control, top) was fused to the WT cytoplasmic domain of ITGα5 (Tat-ITGα5-cyto-WT, middle) and the 5A mutant (Tat-ITGα5-cyto-5A, bottom). **j** Live-cell SIM images of NRK cells expressing TSPAN4-mCherry and ITGα5-GFP, and treated with 100 μM control (Tat), WT (Tat-WT) or mutant (Tat-5A) peptide from **i**. Green, ITGα5; red, TSPAN4; yellow, merge. Scale bar, 10 μm. Boxed regions are enlarged on the right. **k** The boxed regions from **j** were quantified for the ITGα5-GFP fluorescence intensity. **l** Statistical analysis of the number of migrasomes per 100 μm retraction fiber per cell. The original images were captured as in **j**. *n* = 95 for Tat treatment, *n* = 102 for Tat-WT treatment, *n* = 89 for Tat-5A treatment. Data are presented as mean ± SEM; unpaired *t*-test.

Finally, we investigated how Rab35 promotes migrasome formation. Previous literature showed that Rab35 is required for integrin trafficking.^13^ This prompted us to investigate the relationship between integrin and Rab35. Previously we showed that pairing of integrin heterodimers with extracellular matrix (ECM) proteins determines migrasome formation.^5^ Specifically, we found that when cells were grown on a culture dish coated with a specific ECM protein, the integrin heterodimers which can bind to the specific ECM protein are highly enriched in migrasome formation sites and play important roles in migrasome biogenesis. In cells grown on fibronectin, integrin α5β1 is highly enriched in migrasomes. In WT cells, most of the integrin α5 was concentrated on migrasomes, and there was very little integrin α5 in retraction fibers. In contrast, we found that in Rab35 KO cells, instead of concentrating at the migrasome formation sites, ITGα5-GFP was evenly and diffusely distributed along retraction fibers (Fig. 4a–c). This suggests that the targeting of integrin to migrasome formation sites is impaired in the absence of Rab35.

Next, we investigated the molecular mechanism underlying the Rab35-dependent recruitment of ITGα5-GFP. A previous report indicated that all integrin α subunits can associate with Rab21 via the conserved membrane-proximal GFFKR motif, which is also present in integrin α5.^14^ We wondered whether Rab35 can associate with integrin α5 through this motif. To test this hypothesis, we generated an integrin α5 mutant in which GFFKR is mutated to AAAAA (1-5A). As controls, we also generated another 4 mutants in which the 4 successive sets of 5 consecutive amino acids in the cytosolic portion of integrin α5 are mutated to AAAAA (2-5A, 3-5A, 4-5A, 5-5A) (Fig. 4d). Together, these mutants cover the majority of the integrin α5 cytosolic domain. We found that the GFFKR/AAAAA (1-5A) mutant, but not any of the other mutants, showed impaired targeting to migrasome formation sites (Fig. 4e, f; Supplementary information, Fig. S7). This suggests that the GFFKR motif is required for targeting integrin α5 to migrasome formation sites, possibly by affecting the association with Rab35.

Next, we wanted to directly test the possible interaction between Rab35 and integrin α5. Due to the difficulty in reliably detecting interactions involving membrane proteins by immunoprecipitation, we used dual-color fluorescence cross-correlation spectroscopy (dcFCCS) to capture the interaction between Rab35 and the cytosolic domain of integrin α5 (ITGα5-cyto) (Fig. 4g). To perform the assay, we first purified GFP-Rab35-WT and GFP-Rab35-Q67L (constitutively active mutant) proteins. We also synthesized ITGα5-cyto-WT and ITGα5-cyto-1-5A labeled with the fluorophore Cyanine5 (Cy5). When GFP-Rab35-WT was mixed with Cy5-ITGα5-cyto-WT or Cy5 fluorophore, neither of the mixtures exhibited significant cross-correlation signals (Fig. 4h), which indicates no binding between WT Rab35 and Cy5-ITGα5-cyto-WT. However, the constitutively active mutant GFP-Rab35-Q67L exhibited strong cross-correlation signals with Cy5-ITGα5-cyto-WT under similar experimental conditions (Fig. 4h), which indicates that GFP-Rab35-Q67L can bind to Cy5-ITGα5-cyto-WT. In contrast, GFP-Rab35-Q67L had no cross-correlation signals with Cy5-ITGα5-cyto-1-5A (Fig. 4h), which suggests that the GFFKR motif is required for the binding between the cytosolic domain of integrin α5 and the active form of Rab35. It is worth noting that recombinant GFP-Rab35-WT was purified from *E. coli*, and thus should be in the inactive form. As a control, we also tested the binding between GFP-Rab35-S22N and Cy5-ITGα5-cyto-WT. As expected, we found no binding between GFP-Rab35-S22N and Cy5-ITGα5-cyto-WT (Supplementary information, Fig. S8). These data suggest that active Rab35 can bind to the cytosolic domain of integrin α5 through the GFFKR motif.

We reasoned that if Rab35 recruits integrin α5 to migrasome formation sites by interacting with the GFFKR motif of integrin α5, then loading cells with integrin α5-derived peptides containing the GFFKR motif should competitively inhibit the Rab35-mediated recruitment of integrin α5 to migrasome formation sites and reduce migrasome formation. Indeed, we found that treating cells with the plasma membrane-permeable peptide  Tat-ITGα5-cyto-WT reduced both the targeting of integrin to migrasome formation sites and migrasome formation; in contrast, treating cells with the GFFKR/AAAAA mutant peptide failed to block integrin targeting or migrasome formation (Fig. 4i–l). These results suggest that Rab35 promotes migrasome formation by targeting integrin to migrasome formation sites.

### The PI(4,5)P2-Rab35 axis regulates migrasome formation in physiologically relevant settings and is evolutionarily conserved

**Fig. 5:**
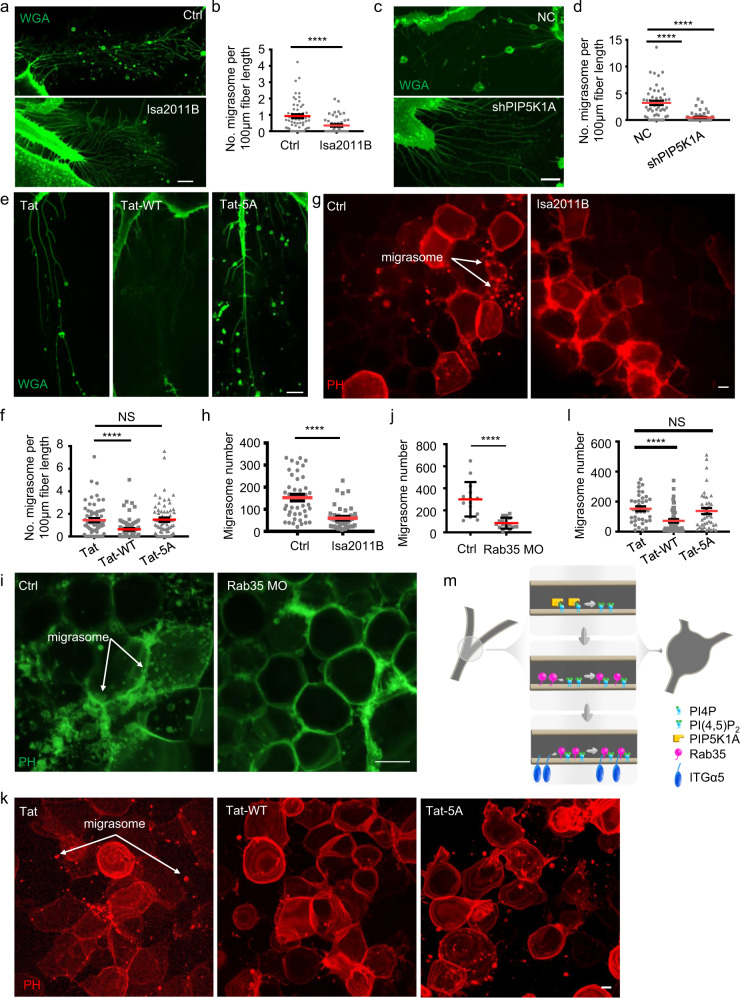
The PI(4,5)P2-Rab35 axis regulates migrasome formation in physiologically relevant settings and is evolutionarily conserved. **a** Live-cell images of BJ cells treated with DMSO (Ctrl) and 20 µM ISA2011B. Green, wheat germ agglutinin (WGA). Scale bar, 10 μm. **b** Statistical analysis of the number of migrasomes per 100 μm retraction fiber per cell. The original images were captured as in **a**. *n* = 59 for Ctrl treatment, *n* = 50 for ISA2011B treatment. Data are presented as mean ± SEM; unpaired *t*-test. **c** Live-cell images of BJ cells treated with negative control (NC) shRNA and shPIP5K1A. Green, WGA. Scale bar, 10 μm. **d** Statistical analysis of the number of migrasomes per 100 μm retraction fiber per cell. The original images were captured as in **c**. *n* = 50 for NC, *n* = 50 for shPIP5K1A. Data are presented as mean ± SEM; unpaired *t*-test. **e** Live-cell images of BJ cells treated with 100 μM control (Tat), WT (Tat-WT) or mutant (Tat-5A) peptide. Green, WGA. Scale bar, 10 μm. **f** Statistical analysis of the number of migrasomes per 100 μm retraction fiber per cell. The original images were captured as in **e**. *n* = 64 for Tat treatment, *n* = 75 for Tat-WT treatment, *n* = 69 for Tat-5A treatment. Data are presented as mean ± SEM; unpaired *t*-test. **g** Live-cell images of gastrulation-stage zebrafish embryos treated with DMSO (Ctrl) and 100 μM ISA2011B. Red, PH-mCherry. Scale bar, 10 μm. **h** Statistical analysis of the number of migrasomes per embryo. The original images were captured as in **g**. *n* = 102 for Ctrl treatment, *n* = 97 for ISA2011B treatment. Data are presented as mean ± SEM; unpaired *t*-test. **i** Live-cell images of gastrulation-stage zebrafish embryos treated with Ctrl and Rab35 MO. Green, PH. Scale bar, 10 μm. **j** Statistical analysis of the number of migrasomes per embryo. The original images were captured as in **i**. *n* = 14 for Ctrl treatment, *n* = 15 for Rab35 MO treatment. Data are presented as mean ± SEM; unpaired *t*-test. **k** Live-cell images of gastrulation-stage zebrafish embryos following injection with 100 μM control (Tat), WT (Tat-WT) or mutant (Tat-5A) peptide at the 8-cell stage. Red, PH-mCherry. Scale bar, 10 μm. **l** Statistical analysis of the number of migrasomes per embryo. The original images were captured as in **k**. *n* = 36 for Tat treatment, *n* = 50 for Tat-WT treatment, *n* = 39 for Tat-5A treatment. Data are presented as mean ± SEM; unpaired *t*-test. **m** Model for the role of the PI(4,5)P2-Rab35 axis in migrasome formation.

Lastly, we tested whether the PI(4,5)P_2_-Rab35 axis regulates migrasome formation in diverse settings. We first tested BJ cells, a fibroblast cell line established from skin taken from normal foreskin of a neonatal male. We found that treating BJ cells with the PIP5K1A inhibitor ISA2011B (Fig. 5a, b), or knocking down PIP5K1A (Fig. 5c, d), significantly impaired migrasome formation. Moreover, treating BJ cells with Tat-ITGα5-cyto-WT, but not the GFFKR/AAAAA mutant peptide, blocked migrasome formation (Fig. 5e, f). These observations suggest that the regulation of migrasome formation by the PI(4,5)P_2_-Rab35 axis is conserved in human cells. Finally, we tested whether the PI(4,5)P_2_-Rab35 axis regulates migrasome formation in vivo. Previously, we reported that migrasomes are formed during zebrafish embryonic development.^2^ Here, we found that treating zebrafish embryos with PIP5K1A inhibitor (Fig. 5g, h) or Tat-ITGα5-cyto-WT peptide, but not the GFFKR/AAAAA mutant peptide (Fig. 5k, l), significantly reduced migrasome formation. Moreover, knockdown of Rab35 using an antisense morpholino oligonucleotide (MO) significantly impaired migrasome formation in zebrafish embryos (Fig. 5i, j; Supplementary information, Fig. S9). Together, these findings suggest that the PI(4,5)P_2_-Rab35 axis regulates migrasome formation in a range of physiological settings and is conserved in different vertebrates.

## Discussion

Our data allow us to propose a provisional model for the signaling events that regulate migrasome biogenesis (Fig. 5m). We propose that the recruitment of PIP5K1A and de novo synthesis of PI(4,5)P_2_ on the migrasome formation site is likely the triggering signal for migrasome formation. Once PI(4,5)P_2_ reaches the concentration threshold, active Rab35 is recruited to the migrasome formation site through its polybasic cluster. Rab35 then serves as an adaptor to recruit integrins to the migrasome formation site. The interaction between active Rab35 and integrin thus creates the necessary adhesion point for migrasome formation.

The biogenesis of organelles is generally tightly regulated by signaling pathways. In many cases, lipid kinases are at the heart of these signaling cascades, which couple metabolic, mechanical and other cues to initiate the biogenesis of a particular organelle. Our results reveal the essential role of the PI(4,5)P_2_-Rab35 axis in migrasome formation. Migrasomes can be added to a growing list of organelles whose biogenesis is controlled by phosphoinositide signaling. Moreover, our data demonstrate that migrasome formation is an active biogenesis process which is tightly regulated by a signaling pathway, rather than a membrane shedding process in which membrane fragments are passively lost from the trailing edge of migrating cells.

We found that PIP5K1A is recruited to the site of migrasome formation prior to formation of migrasomes. At this point, we still do not know how PIP5K1A is recruited to that particular location. It is possible that the recruitment is determined by a specific lipid/protein composition at the migrasome formation site; it is also possible that biophysical properties, such as membrane curvature, may contribute to the preferential recruitment of PIP5K1A. Future study is needed to address this important question.

We observed the rapid accumulation of PI(4,5)P_2_ after recruitment of PIP5K1A, which suggests that at least a proportion of the PI(4,5)P_2_ on migrasome formation sites is synthesized de novo by PIP5K1A located at those sites. The fact that the highly enriched PI(4,5)P_2_ on migrasomes does not diffuse onto the retraction fibers suggests that migrasome formation sites may have unique properties which favor the retention of PI(4,5)P_2_. We speculate that de novo synthesis plus PI(4,5)P_2_ retention may explain the rapid accumulation of PI(4,5)P_2_ on migrasome formation sites.

Our data show that the targeting of integrin α5 to migrasome formation sites is dependent on active Rab35. The dcFCCS analysis indicates that the cytosolic portion of integrin α5 can interact via its GFFKR motif with active Rab35. These data suggest that Rab35 may recruit integrin α5 to the migrasome formation site via direct interaction. It is worth noting that we failed to detect the Rab35/integrin α5 interaction by co-immunoprecipitation/western blotting. We suspect that this stems from the technical difficulty in detecting membrane protein interactions by immunoprecipitation. However, it remains possible that the interaction between Rab35 and integrin α5 is indirect.

Besides integrins, Rab35 is likely to have other effectors and adaptor proteins which contribute to migrasome biogenesis. In addition, it remains unclear how the activity of Rab35 is regulated in the context of migrasome biogenesis. Future investigations to answer these important questions will shed light on the elaborate regulatory network of migrasome biogenesis.

## Materials and methods

### Experimental model and subject details

NRK, MGC803, BJ cells and their derivatives were cultured at 37 °C and 5% CO_2_ in DMEM supplemented by 10% serum, 1% Glutamax and 1% penicillin–streptomycin.

For NRK transfection, one third of cells from a full 6-cm dish were transfected with 3–5 μg plasmid via Amaxa nucleofection using solution T and program NRK. For transfection of MGC803 cells, 70%–90% confluent cultured cells from a 3.5-cm dish were transfected with 5 μg DNA via a Lipofectamine 3000 transfection kit (Invitrogen).

All WT zebrafish used in this study were from the Tuebingen (Tu) strain.

### Constructs

For all the PI(4,5)P_2_-binding proteins identified in the MS screen, the corresponding genes were cloned from rat cDNA and transferred into pmCherry-C1 and -N1. GFP-PIP5K1A, GFP-PLCD3, GFP-Rab35 and their derivatives were cloned into pB-GAG-BGH. PLCγ-PH-GFP was cloned into pEGFP-N1. ITGα5 was described before.^5^ ITGα5 mutations were generated from original plasmid. His-GFP-Rab35 and His-GFP-Rab35-Q67L were cloned into pET21b.

### Generation of KO cell lines

To generate KO cell lines, the PIP5K1A, PLCD3 and Rab35 coding genes in NRK cells were deleted by a modified PX458 plasmid as described before.^6^ The sgRNA sequences were:

PIP5K1A-gRNA-1-F: 5′-CACC GGATAAACAGGCAGTGGCTG-3′

PIP5K1A-gRNA-1-R: 5′-AAAC CAGCCACTGCCTGTTTATCC-3′

PIP5K1A-gRNA-2-F: 5′-CACC GAGTTGGTGGAGGCTAAGGG-3′

PIP5K1A-gRNA-2-R: 5′-AAAC CCCTTAGCCTCCACCAACTC-3′

PLCD3-gRNA-1-F: 5′-CACC GTTCGCCCCTGCTAGTGAGT-3′

PLCD3-gRNA-1-R: 5′-AAAC ACTCACTAGCAGGGGCGAAC-3′

PLCD3-gRNA-2-F: 5′-CACC GCACCAAAAGGCCCGGGCTA-3′

PLCD3-gRNA-2-R: 5′-AAAC TAGCCCGGGCCTTTTGGTGC-3′

Rab35-gRNA-1-F: 5′-CACC GGCGACCAGGGTGCACCCCA-3′

Rab35-gRNA-1-R: 5′-AAAC TGGGGTGCACCCTGGTCGCC-3′

Rab35-gRNA-2-F: 5′-CACC GGAGGCGGTGCGGGCCCTGC-3′

Rab35-gRNA-2-R: 5′-AAAC GCAGGGCCCGCACCGCCTCC-3′

After 48 h transfection, cells were sorted for GFP signal by fluorescence-activated cell sorting and seeded into 96-well plates. For PIP5K1A and Rab35, KO clones were identified by western blotting. For PLCD3, KO clones were identified by PCR, which yielded a smaller product. Primers for PLCD3 knockout identification were:

ID-PLCD3-F: 5′-GTCAGAATTCCCAGAAAAAAGTGTCTGC-3′

ID-PLCD3-R: 5′-GAAGCCAGTTAGCCCGTACACC-3′

### Immunofluorescence

Cells were washed with phosphate buffered saline (PBS), and then fixed in medium: 4% paraformaldehyde (1:1) for 5 min, followed by fixation in 4% paraformaldehyde for 5 min. Fixed cells were permeabilized and blocked in 0.05% saponin, 10% FBS in PBS for 30 min, stained with antibody according to the manufacturer’s instructions in 10% FBS in PBS at 4 °C overnight, and washed with PBS three times. Cells were stained with secondary antibody in 10% FBS in PBS for 1 h and washed with PBS three times.

### Imaging and image analysis

Confocal snapshot images were acquired using a Fluoview 1000 confocal microscope (Olympus), and a NIKON A1. Images were collected at 1024 × 1024 pixels. Long-term time-lapse images of living cells were collected using a NIKON A1 microscope. Images were collected at 1024 × 1024 pixels. SIM snapshot images were collected by SIM set up on the NIKON A1. Fluorescence intensities of snapshot images were analyzed using ImageJ Fiji, and statistical analyses were conducted using GraphPad Prism 7. Fluorescence intensities of long-term time-lapse images were statistically analyzed by NIS-element analysis software.

To acquire two-dimensional images and two-dimensional live images of embryos, mRNA was injected at the desired embryonic stage. The embryos were then embedded in 1% low-melting point agarose and imaged by Dragonfly spinning disk microscopy.

### Acquisition of a candidate list for migrasome-enriched PI(4,5)P_2_-binding proteins

Gene Ontology (GO) term of PI(4,5)P_2_ binding (GO:0005546) was from The Gene Ontology knowledgebase http://amigo.geneontology.org/amigo/term/GO:0005546.

The intersection of this list and list of proteins enriched on migrasomes identified by MS are migrasome-enriched PI(4,5)P2-binding proteins.

### Protein purification

pET21b-His-GFP-Rab35 was expressed in *E. coli* BL21 (DE3) cells cultured at 16 °C for 18 h with induction by isopropyl-β-D-thiogalactoside (IPTG) at a final concentration of 0.2 mM. His-GFP-Rab35 was purified by Ni^2+^-NTA agarose affinity chromatography in buffer containing 20 mM HEPES, pH 8.0, 100 mM NaCl, 2 mM MgCl_2_, 1 mM DTT and protease inhibitor cocktail.

### Peptide synthesis

The sequences of peptides from the integrin α5 cytoplasmic region were

WT: YKLGFFKRSLPYGTAMEKAQLKPPATSDA

5A: YKLAAAAASLPYGTAMEKAQLKPPATSDA

Tat-WT: YGRKKRRQRRRGGYKLGFFKRSLPYGTAMEKAQLKPPATSDA

Tat-5A: YGRKKRRQRRR GGYKLAAAAASLPYGTAMEKAQLKPPATSDA

Tat: YGRKKRRQRRRGG

Peptides were synthesized by Synpeptide Co., Ltd.

### ITGα5-cyto peptide labeling

Synthesized WT or mutant ITGα5-cyto peptide was mixed with Sulfo-Cy5 NHS ester (Lumiprobe) at 1:1 molar ratio in reaction buffer (50 mM HEPES, pH 7.5, 100 mM NaCl, 2 mM MgCl_2_) and incubated for 2 h at 25 °C. For the control, Tris (pH 8.0) was mixed with Sulfo-Cy5 NHS ester at 7:1 molar ratio in reaction buffer, as ITGα5-cyto-WT peptide has 7 -NH_2_ groups. To fully quench the reaction, 20-fold Tris (pH 8.0) was added to react with the excess free dye for 20 min and then the mixture was centrifuged at 13,000 rpm for 10 min to remove the precipitate. The supernatant was used for the following dcFCCS assay.

### dcFCCS measurements and data analysis

dcFCCS measurements were conducted on a home-built confocal microscope, based on a Zeiss AXIO Observer D1 fluorescence microscope equipped with solid-state 488 nm and 640 nm excitation lasers (Coherent Inc. OBIS Smart Lasers), an oil-immersion objective (Zesis, 100×, numerical aperture = 1.4) and avalanche photodiode detectors (APDs, Excelitas, SPCM-AQRH-14) as previously described.^15^ Fluorescence passed through a pinhole (50 μm diameter), and then was split by a T635lpxr dichroic mirror (Chroma). Bandpass filters ET525/50m (Chroma) and ET700/75m (Chroma) were used to further filter fluorescence for GFP and Cy5 detection channels, respectively.

WT or mutated GFP-Rab35 protein was centrifuged at 13,000 rpm for 10 min to remove aggregates. dcFCCS experiments were carried out with 488 nm and 640 nm laser excitation at 25 °C. GFP-Rab35 and ITGα5-cyto were mixed in 20 mM HEPES, pH 7.5, 100 mM NaCl, 2 mM MgCl_2_, 0.1% BSA and then loaded immediately onto coverslips passivated with polyethylene glycol. Raw data of photon arrival time were recorded for 5 min. Experiments were repeated three times for each experimental condition.

### Zebrafish mRNA, peptide and MO injection

For live-cell imaging, one cell of a zebrafish embryo at the eight-cell stage was injected with 100 pg PH-mCherry mRNA. For peptide injection, 1 nL of 1 mM Tat, Tat-WT or Tat-5A was injected into the yolk at the eight-cell stage. For antisense MO injection, 10 ng was injected into the yolk at the one-cell stage. Confocal images were taken at 6 hpf. Antisense MOs were obtained from GeneTools LLC and resuspended in MilliQ H_2_O to give a stock concentration of 20 μg/μL. For the knockdown of Rab35a, an MO was designed with the following sequence (Rab35a AUG): 5′-ACAGGTAATCATAATCCCGCGCCAT-3′.

## Supplementary information

Supplementary information, Fig. S1

Supplementary information, Fig. S2

Supplementary information, Fig. S3

Supplementary information, Fig. S4

Supplementary information, Fig. S5

Supplementary information, Fig. S6

Supplementary information, Fig. S7

Supplementary information, Fig. S8

Supplementary information, Fig. S9

Supplementary information, Video S1

Supplementary information, Video S2

Supplementary information, Video S3

Supplementary information, Video S4

Supplementary information, Video S5

Legends for Supplementary Videos
