# Supplementary material for: The phosphatidylinositol (4,5)-bisphosphate-Rab35 axis regulates migrasome formation
Source: Cell Res. 2023 May 4;33(8):617–27. doi: 10.1038/s41422-023-00811-5 (PMC10397319; doi:10.1038/s41422-023-00811-5)
Supplement: Supplementary file 1 — Supplementary information, Fig. S1 [file 41422_2023_811_MOESM1_ESM.pdf]

a

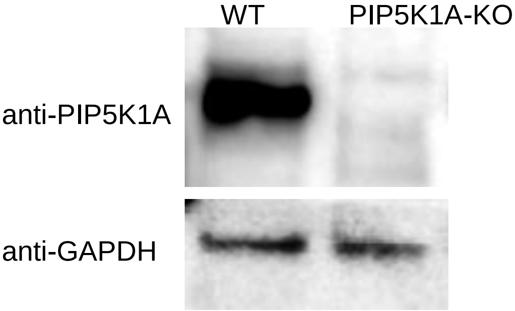

b

WT：

CTAGACTGAGCGCCGAGAAGGTGGTGCGCATGCGCAACACCGGGGTTTCCTTGGCCGCCCACCCTGAACGAA
GCTGTGGAGTTGGGGATAAACAGGCAGTGGCTGGGGAGGAGAGGGAGGGGGGCATTCGCTTTTTGGGGGACC
TCAGGTGCCTGGGTTTTTCTTTAGGTTTGTTCTATCCGAGACGCGGGACCCCCGGGGCCCGGCTTGAGTGTTCC
TGTCTAGTGAGAGAAGGTAGCTTTTGCGGAGAGCAGTTCAAGTTGCTAGGAAGGTGCCAGACTGGATCTCTCCC
CCATGCCGGGCAAGTGGTGCGGTCCTGAGCTTGTCCTGGAGCCTGAACAACGCCTGTGAGAAGCCCGCCGAAC
GCGGGTTCTGTGAAGAGACGTGGGGAAGATTCGATTCCGAGAAAAGGAAAAGTCGGATTGAAAGGGAGTGAAG
CCGCTGAGGGGGAGGGGGCTGCCAAGATGGCGTCCGCCTCCTCAGGGCCAGCGGCGGCGGGGTTTTCACCC
CTTGATTCCGGGGTCCCTGCCGGTACCGCAGGTGAGTCGGGTAGGGCGCGGGCAGACTGCTGGGGAGGACTG
ACTGTGAGATGGGAACAAGAGCGAGTCTTGGGAAAGGACCTGTAGCTGAGGATGCCCAAGGGGTTGCCCCTAA
GGGAGGACAGGACCGGAATTTGAAGTAAAAGACAGTTCTATTACTAGAGGAGTCTGGGGGAAAAGGATGGATGT
CACTGAGAAAAGGGAAGCTGACGAAAACTCACACAGTGTCTTAGAGGCGACTGCGTTGAGAACTTTGAGGGGG
AAGAATGGAGATTGAAATTGAAGGTTAGGGGTTAAGAGAGAAACCACAAAAACAGGAGTGGGAAACTAGAGTTG
GTGGAGGCTAAGGGAGGAGACAGCTAGGTCTTGCCTAGCATGCATCAGGATCTGGGCTCAATAAGACAAAAAA
GTAAATGGAGTATTGGTAGAGGGGAATTCCTTGGTATAAGGTAGAGGCCAAGGTGACTGT

PIP5K1A-KO：

CTAGACTGAGCGCCGAGAAGGTGGTGCGCATGCGCAACACCGGGGTTTCCTTGGCCGCCCACCCTGAACGAA
GCTGTGGAGTTGGGGATAAACAGGCAGTGGGACAGGACCGGAATTTGAAGTAAAAGACAGTTCTATTACTAGAG
GAGTCTGGGGGAAAAGGATGGATGTCACTGAGAAAAGGGAAGCTGACGAAAACTCACACAGTGTCTTAGAGGC
GACTGCGTTGAGAACTTTGAGGGGGAAGAATGGAGATTGAAATTGAAGGTTAGGGGTTAAGAGAGAAACCACAA
AAACAGGAGTGGGAAACTAGAGTTGGTGGAGGCTAAGGGAGGAGACAGCTAGGTCTTGCCTAGCATGCATCAG
GATCTGGGCTCAATAAGACAAAAAAGTAAATGGAGTATTGGTAGAGGGGAATTCCTTGGTATAAGGTAGAGGCC
AAGGTGACTG

a PIP5K1A knockout efficiency was analyzed by western blotting with antibodies against PIP5K1A and GAPDH.

b Sequence of the PIP5K1A gene in WT and PIP5K1A-KO NRK cells. The KO cell line lacks the bases highlighted in green. The deleted sequence includes the initiation codon of PIP5K1A (ATG, pink).
