# Supplementary material for: The phosphatidylinositol (4,5)-bisphosphate-Rab35 axis regulates migrasome formation
Source: Cell Res. 2023 May 4;33(8):617–27. doi: 10.1038/s41422-023-00811-5 (PMC10397319; doi:10.1038/s41422-023-00811-5)
Supplement: Supplementary file 2 — Supplementary information, Fig. S2 [file 41422_2023_811_MOESM2_ESM.pdf]

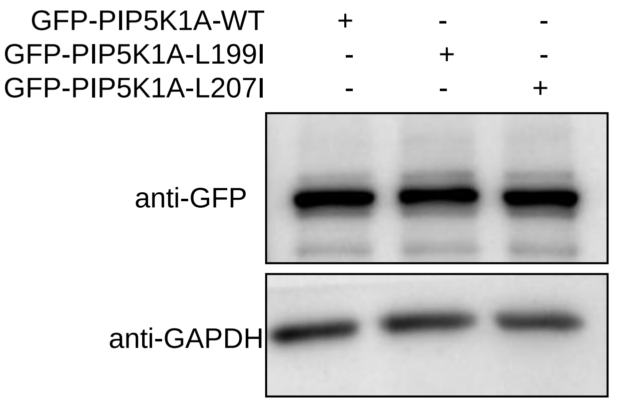

Western blot showing comparable expression levels of PIP5K1A-WT, PIP5K1A-L199I and PIP5K1A-L207I in PIP5K1A-KO NRK cells. Proteins were detected with antibodies against GFP and GAPDH.
