# Supplementary material for: The phosphatidylinositol (4,5)-bisphosphate-Rab35 axis regulates migrasome formation
Source: Cell Res. 2023 May 4;33(8):617–27. doi: 10.1038/s41422-023-00811-5 (PMC10397319; doi:10.1038/s41422-023-00811-5)
Supplement: Supplementary file 3 — Supplementary information, Fig. S3 [file 41422_2023_811_MOESM3_ESM.pdf]

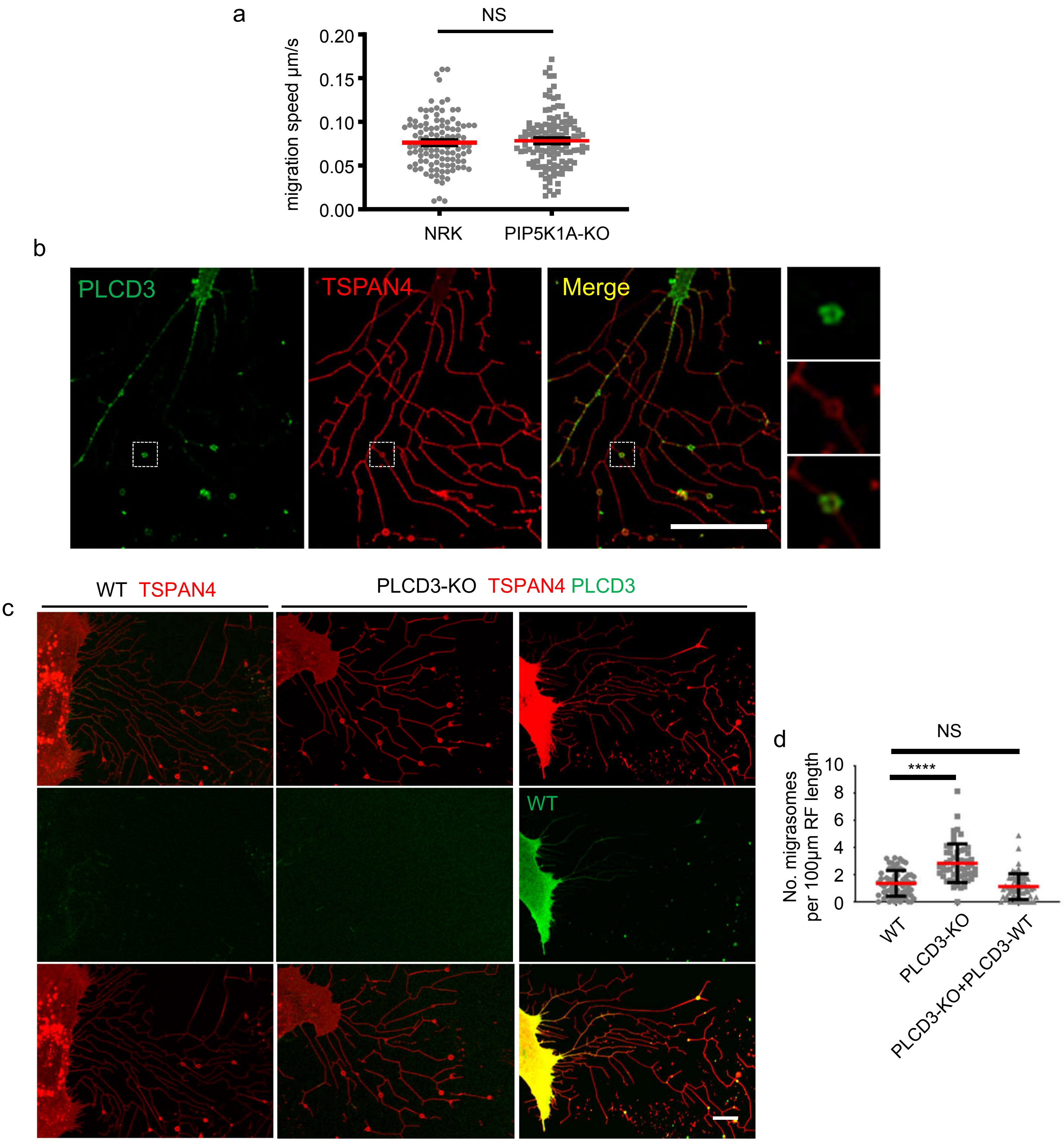
a
NS
migration speed µm/s
0.20
0.15
0.10
0.05
0.00
NRK
PIP5K1A-KO
b
PLCD3
TSPAN4
Merge
c
WT TSPAN4
PLCD3-KO TSPAN4 PLCD3
WT
d
NS
****
No. migrasomes per 100µm RF length
10
8
6
4
2
0
WT
PLCD3-KO
PLCD3-KO+PLCD3-WT

a Quantification of the migration speed of WT and PIP5K1A KO NRK cells. Mean ± s.e.m., unpaired t-test.
b Live-cell confocal microscopy images of NRK cells expressing PLCD3-GFP and TSPAN4-mCherry. Green, PLCD3; red, TSPAN4; yellow, merge. Scale bar, 10 μm. Boxed regions are enlarged at the right.
c Live-cell images of WT NRK-TSPAN4-mCherry cells, PLCD3-KO NRK-TSPAN4-mCherry cells, and PLCD3-KO NRK-TSPAN4-mCherry cells with rescue by GFP-PLCD3. Green, PLCD3; red, TSPAN4; yellow, merge. Scale bar, 10 μm.
d The images from **c** were quantified for the migrasome number per 100 μm fiber per cell. Mean ± S.D.. ****$P < 0.0001$; NS, not significant.
