# Supplementary material for: The phosphatidylinositol (4,5)-bisphosphate-Rab35 axis regulates migrasome formation
Source: Cell Res. 2023 May 4;33(8):617–27. doi: 10.1038/s41422-023-00811-5 (PMC10397319; doi:10.1038/s41422-023-00811-5)
Supplement: Supplementary file 4 — Supplementary information, Fig. S4 [file 41422_2023_811_MOESM4_ESM.pdf]

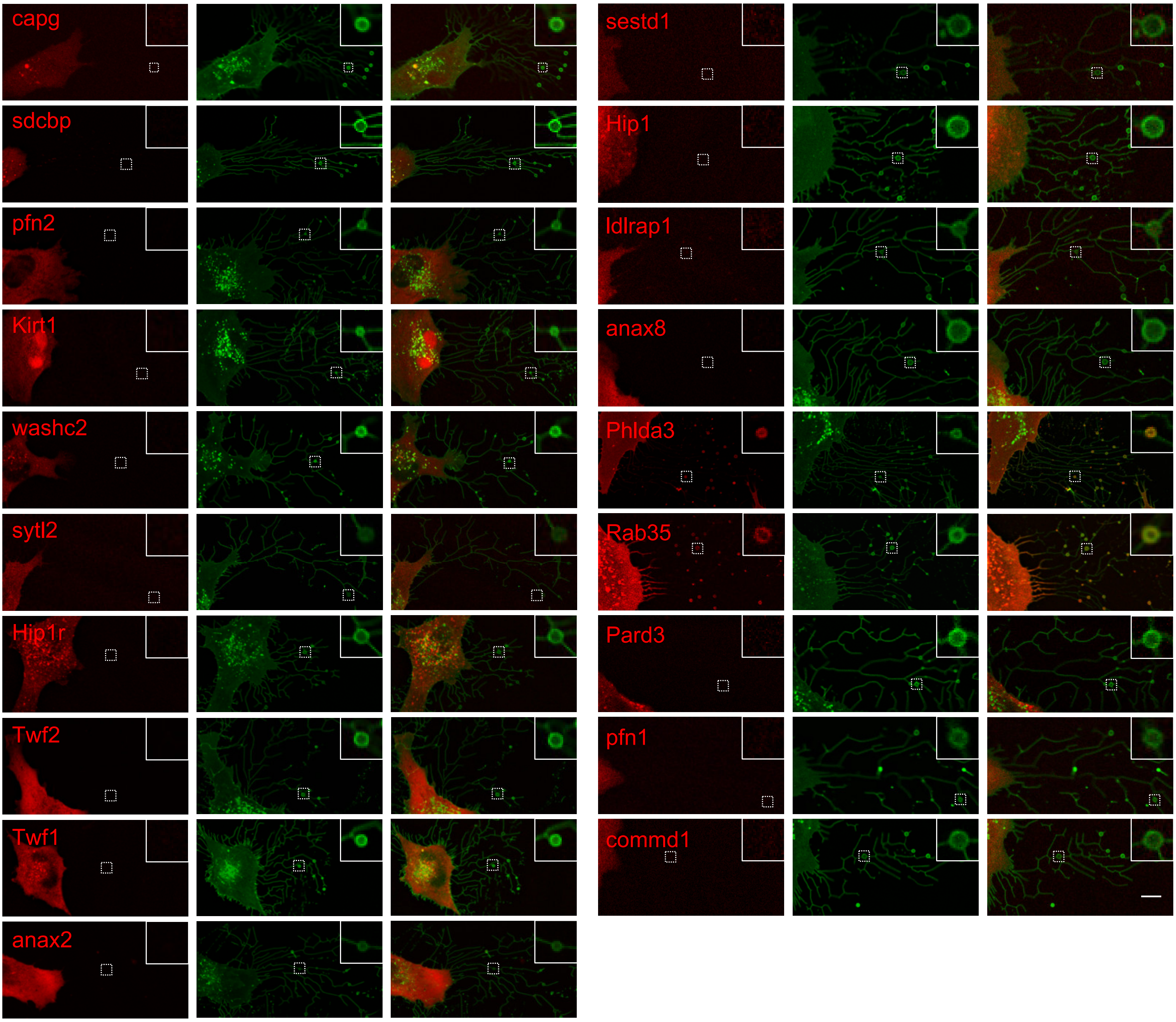

Live-cell confocal microscopy images of NRK cells expressing TSPAN4-GFP and mCherry-tagged PIP_2-binding proteins. Green, TSPAN4; red, PIP_2-binding proteins; yellow, merge. Scale bar, 10 μm. Inserts show enlarged migrasomes.
