# Supplementary material for: The phosphatidylinositol (4,5)-bisphosphate-Rab35 axis regulates migrasome formation
Source: Cell Res. 2023 May 4;33(8):617–27. doi: 10.1038/s41422-023-00811-5 (PMC10397319; doi:10.1038/s41422-023-00811-5)
Supplement: Supplementary file 5 — Supplementary information, Fig. S5 [file 41422_2023_811_MOESM5_ESM.pdf]

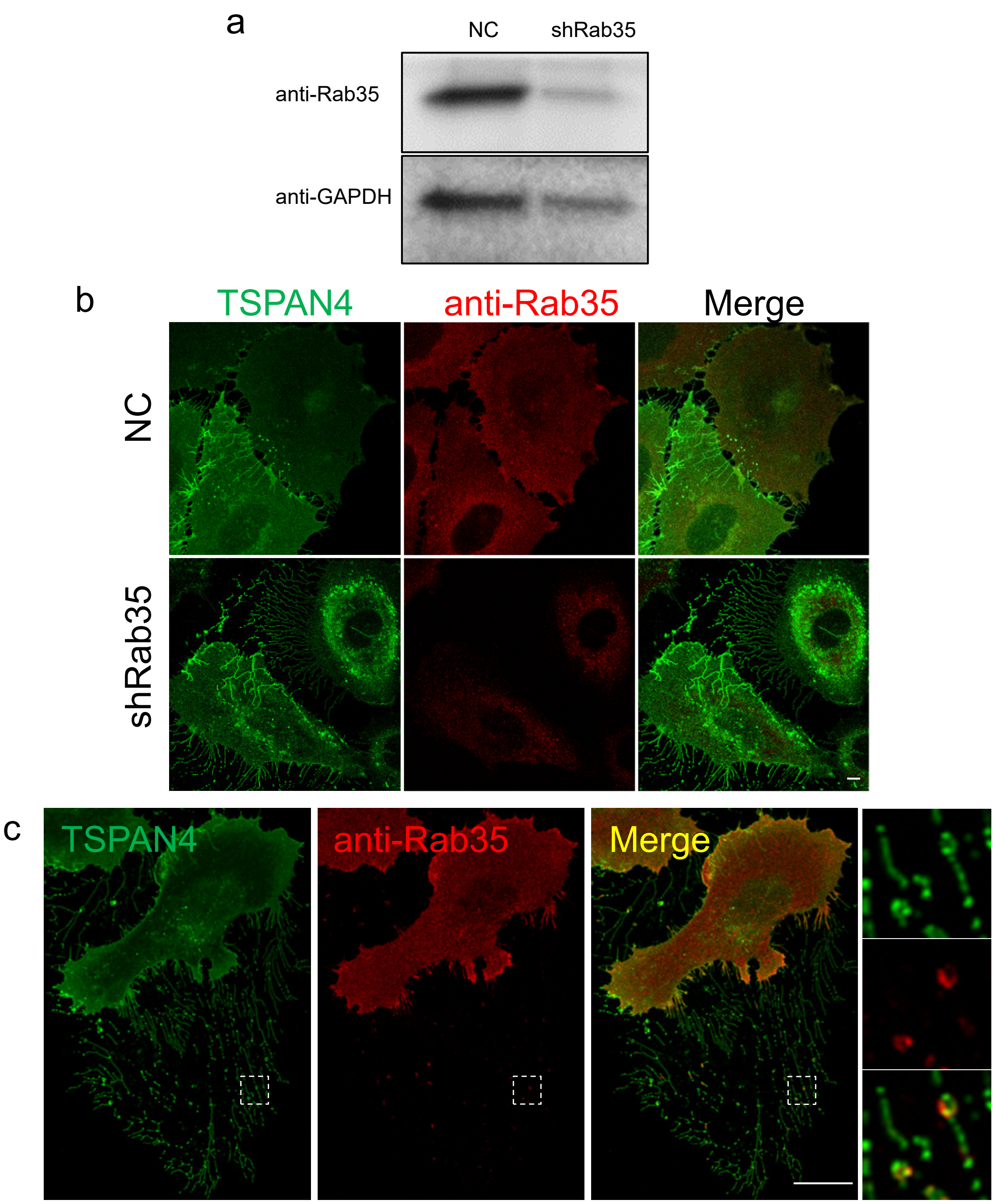

a Rab35 knockdown efficiency was analyzed by western blotting with antibodies against Rab35 and GAPDH.
b Immunofluorescence imaging of Rab35 in WT and shRab35 NRK TSPAN4-mCherry cells. Scale bar, 10 µm.
c Immunofluorescence imaging of Rab35 in NRK TSPAN4-mCherry cells. Green, TSPAN4; red, Rab35; yellow, merge. The boxed areas are enlarged at the right. Scale bar, 10 µm.
