# Supplementary material for: The phosphatidylinositol (4,5)-bisphosphate-Rab35 axis regulates migrasome formation
Source: Cell Res. 2023 May 4;33(8):617–27. doi: 10.1038/s41422-023-00811-5 (PMC10397319; doi:10.1038/s41422-023-00811-5)
Supplement: Supplementary file 6 — Supplementary information, Fig. S6 [file 41422_2023_811_MOESM6_ESM.pdf]

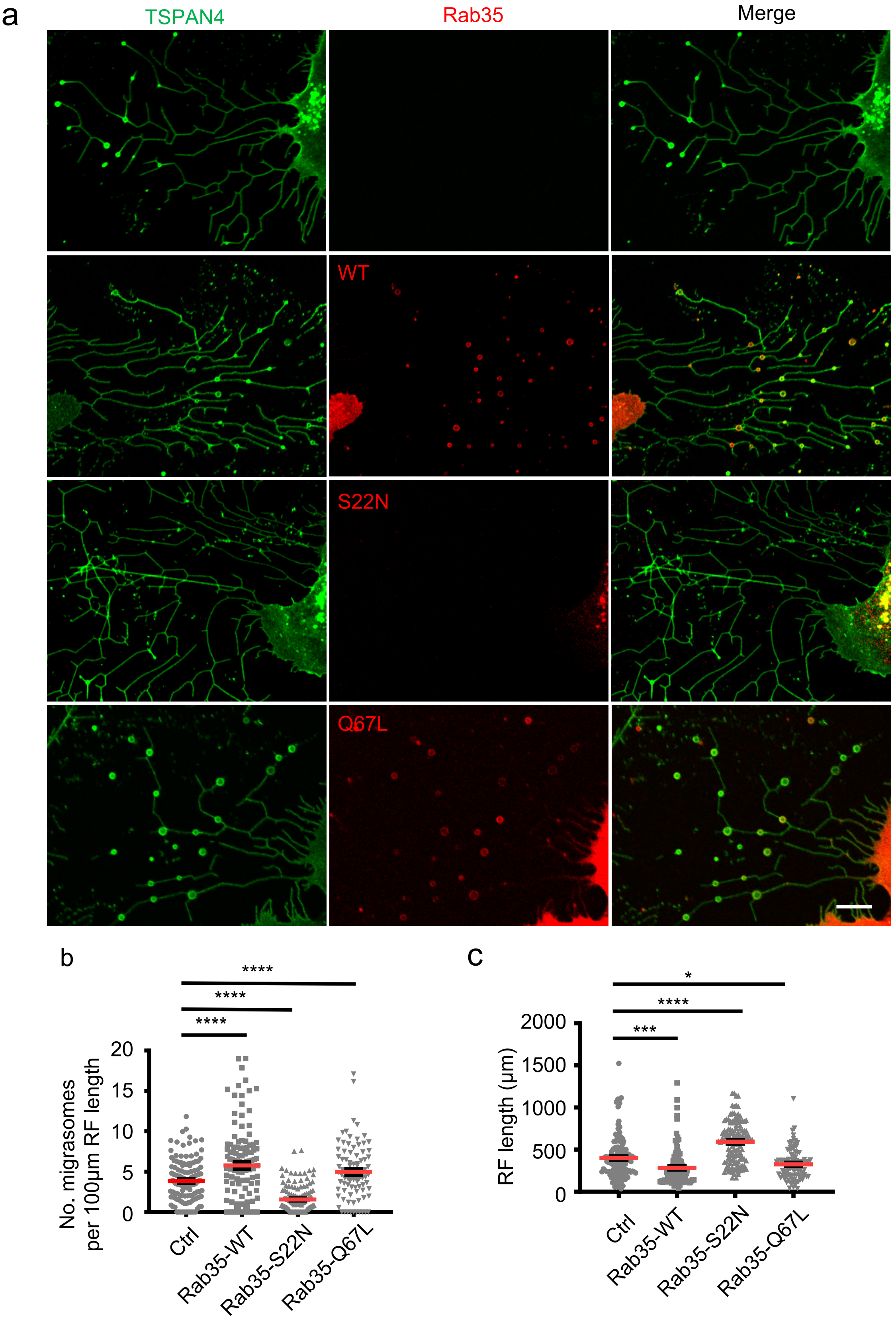
a
TSPAN4
Rab35
Merge
WT
S22N
Q67L
b
****
****
****
No. migrasomes per 100μm RF length
20
15
10
5
0
Ctrl
Rab35-WT
Rab35-S22N
Rab35-Q67L
c
*
****
***
RF length (μm)
2000
1500
1000
500
0
Ctrl
Rab35-WT
Rab35-S22N
Rab35-Q67L

a Live-cell confocal microscopy images of NRK-TSPAN4-GFP cells and NRK-TSPAN4-GFP cells stably expressing mCherry-Rab35-WT, mCherry-Rab35-S22N or mCherry-Rab35-Q67L. Green, TSPAN4; red, Rab35; yellow, merge. Scale bar, 10 μm.
b Statistical analysis of the number of migrasomes per 100 μm retraction fiber per cell. The original images were captured as in **a**. n=133 for NRK; n=114 for NRK+Rab35-WT; n=140 for NRK+Rab35-S22N; n=89 for NRK+Rab35-Q67L. Mean ± s.e.m., unpaired t-test.
c Statistical analysis of retraction fiber length per cell. The original images were captured as in **a**. n=133 for NRK; n=114 for NRK+Rab35-WT; n=136 for NRK+Rab35-S22N; n=89 for NRK+Rab35-Q67L. Mean ± s.e.m., unpaired t-test.
