# Supplementary material for: The phosphatidylinositol (4,5)-bisphosphate-Rab35 axis regulates migrasome formation
Source: Cell Res. 2023 May 4;33(8):617–27. doi: 10.1038/s41422-023-00811-5 (PMC10397319; doi:10.1038/s41422-023-00811-5)
Supplement: Supplementary file 7 — Supplementary information, Fig. S7 [file 41422_2023_811_MOESM7_ESM.pdf]

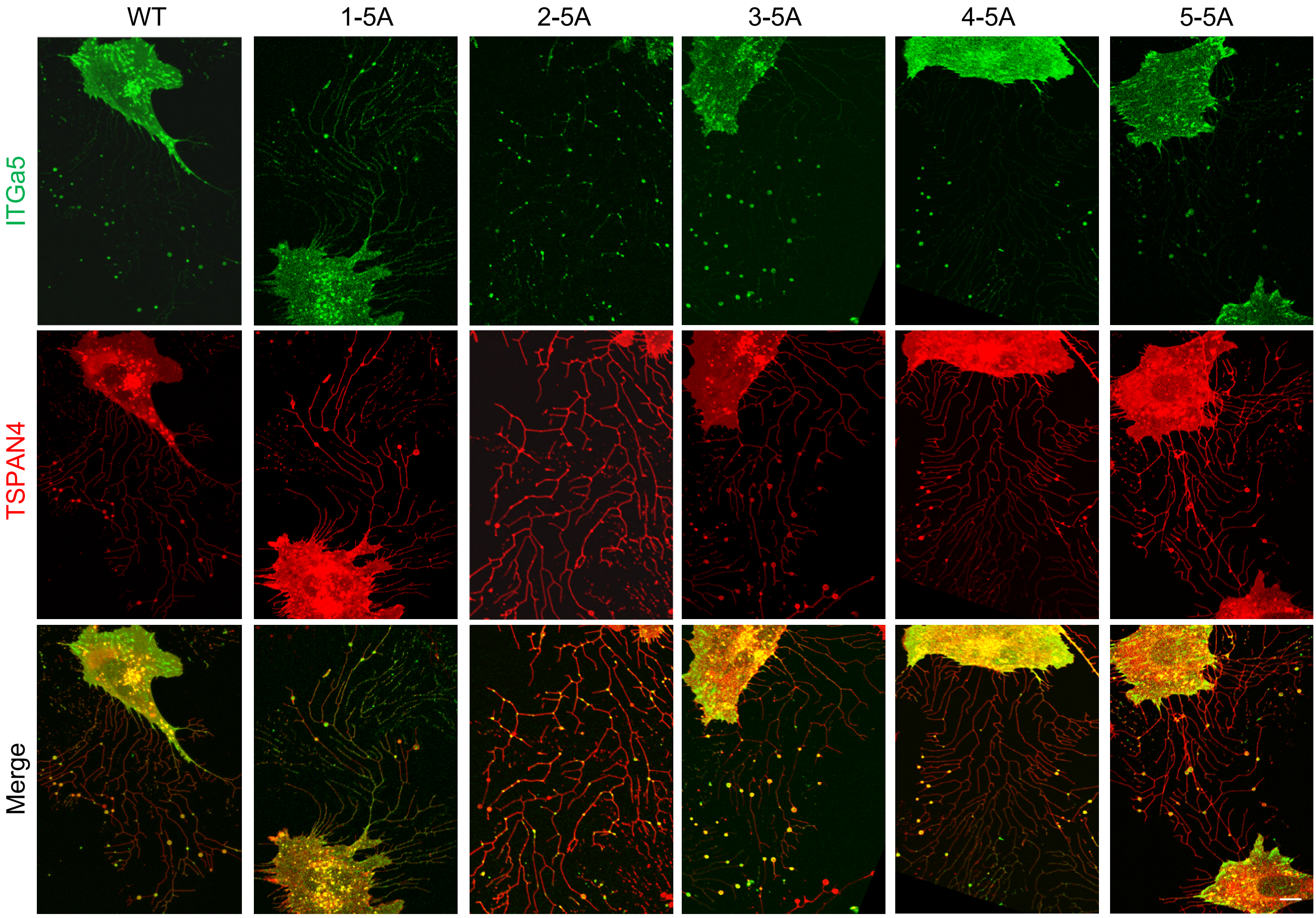

Live-cell confocal microscopy images of NRK-TSPAN4-mCherry cells expressing ITGα5-GFP WT and cytosolic domain mutants. Green, ITGα5; red, TSPAN4; yellow, merge. Scale bar, 10 μm.
