# Supplementary material for: The phosphatidylinositol (4,5)-bisphosphate-Rab35 axis regulates migrasome formation
Source: Cell Res. 2023 May 4;33(8):617–27. doi: 10.1038/s41422-023-00811-5 (PMC10397319; doi:10.1038/s41422-023-00811-5)
Supplement: Supplementary file 8 — Supplementary information, Fig. S8 [file 41422_2023_811_MOESM8_ESM.pdf]

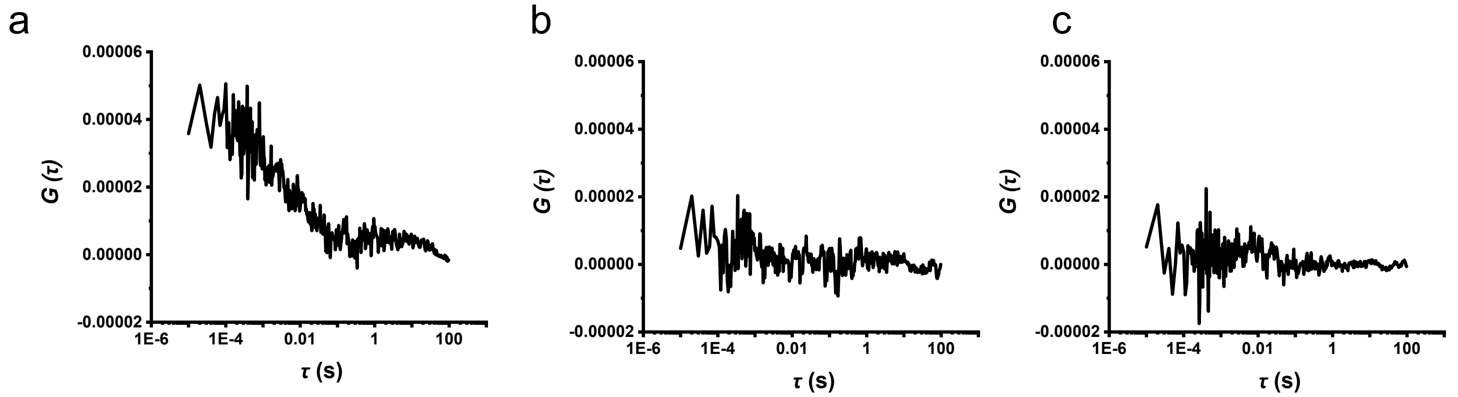

dcFCCS curves of 100 nM GFP-Rab35-Q67L mixed with 500 nM Cy5-ITGα5-cyto-WT **(a);** 100 nM GFP-Rab35-Q67L mixed with 500 nM Cy5-ITGα5-cyto-5A **(b)**; and 100 nM GFP-Rab35-S22N mixed with Cy5-ITGα5-cyto-WT **(c)**.
