# Supplementary figures and images for: The phosphatidylinositol (4,5)-bisphosphate-Rab35 axis regulates migrasome formation
Source: Cell Res. 2023 May 4;33(8):617–27. doi: 10.1038/s41422-023-00811-5 (PMC10397319; doi:10.1038/s41422-023-00811-5)

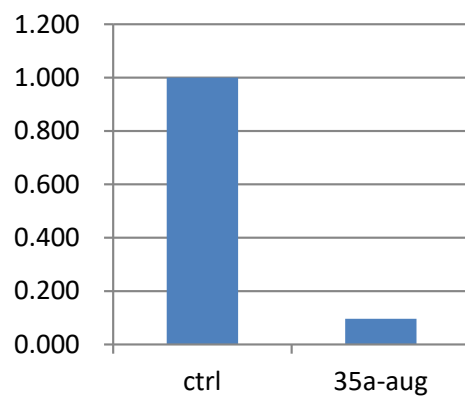

Knockdown efficiency of Rab35 MO quantified by qPCR.

Supplement: Supplementary file 9 — Supplementary information, Fig. S9 [file 41422_2023_811_MOESM9_ESM.pdf]
