# Supplementary material for: The phosphatidylinositol (4,5)-bisphosphate-Rab35 axis regulates migrasome formation
Source: Cell Res. 2023 May 4;33(8):617–27. doi: 10.1038/s41422-023-00811-5 (PMC10397319; doi:10.1038/s41422-023-00811-5)
Supplement: Supplementary file 15 — Legends for Supplementary Videos [file 41422_2023_811_MOESM15_ESM.pdf]

Supplementary Video 1

Movie of an NRK cell expressing PH-GFP and TSPAN4-mCherry. Images were collected by SIM at 1 frame/5 min. Scale bar, 10 μm.

Supplementary Video 2

Movie of an NRK cell expressing PIP5K1A-GFP and TSPAN4-mCherry. Images were collected by SIM at 1 frame/8 min. Scale bar, 10 μm.

Supplementary Video 3

Movie showing the migration of WT NRK-TSPAN4-mCherry cells. Images were collected with a confocal microscope at 1 frame/6 min. Scale bar, 20 μm.

Supplementary Video 4

Movie showing the migration of PIP5K1A-KO NRK-TSPAN4-mCherry cells. Images were collected with a confocal microscope at 1 frame/6 min. Scale bar, 20 μm.

Supplementary Video 5

Movie of an NRK cell expressing TSPAN4-GFP and mCherry-Rab35. Images were collected by SIM at 1 frame/5 min. Scale bar, 10 μm.
